# Critical transition between cohesive and population-dividing responses to change

**DOI:** 10.1098/rsif.2012.0431

**Published:** 2012-07-18

**Authors:** Rachata Muneepeerakul, Murad R. Qubbaj, Rimjhim M. Aggarwal, John M. Anderies, Marco A. Janssen

**Affiliations:** 1 School of Sustainability, Arizona State University, Tempe, AZ 85287, USA; 2 Mathematical, Computational, and Modeling Sciences Center, Arizona State University, Tempe, AZ 85287, USA; 3 School of Human Evolution and Social Change, Arizona State University, Tempe, AZ 85287, USA

**Keywords:** population dividing, critical transition, replicator dynamics

## Abstract

Globalization and global climate change will probably be accompanied by rapid social and biophysical changes that may be caused by external forcing or internal nonlinear dynamics. These changes often subject residing populations (human or otherwise) to harsh environments and force them to respond. Research efforts have mostly focused on the underlying mechanisms that drive these changes and the characteristics of new equilibria towards which populations would adapt. However, the transient dynamics of how populations respond under these new regimes is equally, if not more, important, and systematic analysis of such dynamics has received less attention. Here, we investigate this problem under the framework of replicator dynamics with fixed reward kernels. We show that at least two types of population responses are possible—cohesive and population-dividing transitions—and demonstrate that the critical transition between the two, as well as other important properties, can be expressed in simple relationships between the shape of reward structure, shift magnitude and initial strategy diversity. Importantly, these relationships are derived from a simple, yet powerful and versatile, method. As many important phenomena, from political polarization to the evolution of distinct ecological traits, may be cast in terms of division of populations, we expect our findings and method to be useful and applicable for understanding population responses to change in a wide range of contexts.

## 1. Introduction

Over the past decade, we have witnessed a series of rapid and unprecedented changes at the global scale. The food crisis of 2007 and subsequently the financial crisis of 2008, the Arab Spring and the European debt crisis in 2011 have paved the way for major restructuring of the political, economic and social systems around the world. Such rapid social changes, punctuated by periods of stability, are also well represented in the historical and archaeological records [1]. Recent work in ecology and earth system science suggests that natural systems, too, exhibit rapid shifts [2,3]. The underlying causes of these shifts, be they external forcings or internal nonlinear dynamics [4,5], have deservedly received much attention. This has been accompanied by much discussion that focuses on what new configurations, or equilibria, populations residing in such changing environments would adapt towards. However, understanding *how* populations respond under these new environments or regimes—i.e. the transient dynamics—is equally, if not more, important [6]. Given the adaptive nature of populations, the transient dynamics may play a crucial role in determining the very characteristics of the new equilibria. For example, if a population splits into groups as it responds to an exogenously imposed change, this may lead to potentially costly internal conflict and jeopardize the possibility of the population actually reaching the new equilibrium. Understanding the dynamics of such population responses is the focus of this paper. This focus on the transient behaviour—as opposed to the endpoint equilibrium—provides an important complementary perspective to help investigate problems related to population responses to change. Particularly, we ask: What types of transitions in populations, human or otherwise, can be induced by rapid shifts in the biophysical and social environment?

To investigate these transition dynamics, we consider a simple model in which the environment shifts suddenly and a population of agents characterized by a continuous distribution of strategies (or traits) respond to this shift. We assume that before the shift, the population had been exposed to a particular set of environmental conditions—a regime—for an extended period of time. The population would have therefore adapted in the sense that agents have fine-tuned their strategies to fit that regime, and consequently performed rather well. A shift then occurs. Compared with the performance just prior to the shift, the population's overall performance initially plummets, but subsequently recovers through an adaptive process involving changes in the strategy distribution. Broadly speaking, the preceding description characterizes many social and ecological systems, especially in this era of globalization and global climate change [7–9].

## 2. The model

Such scenarios of shifts and responses can be studied through the so-called replicator equation [10–14]. The continuous replicator equation is defined as2.1
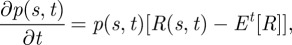
where *p*(*s, t*) is the probability density function (pdf), or frequency distribution, of strategy *s* at time *t*, *R*(*s, t*) the ‘reward kernel’ specifying the reward earned by users of strategy *s* at time *t* (depending on the context, ‘reward’ may mean actual monetary reward, fitness, reproductive success, etc.), and *E*^*t*^[*R*] = ∫*p*(*s,t*)*R*(*s,t*)d*s* the population-averaged reward at time *t* [12,14,15]. Equation (2.1) describes how *p*(*s, t*) evolves, driven by a ubiquitous feature observed in many systems: if a strategy performs better than the average, its frequency increases, and vice versa. In social systems, this effect can be generated through social learning—agents copy strategies that perform better than average; in ecological systems, this simply reflects higher reproductive fitness of users of better strategies. Recent work on this equation [13,14] has shown that its solution takes the form of time-dependent Boltzmann distribution:2.2
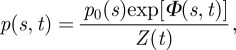
where *p*_0_(*s*) = *p*(*s*,0), *Φ*(*s,t*) = ∫_0_^*t*^ *R*(*s*,*τ*)d*τ* and *Z*(*t*) = ∫*p*_0_(*s*)exp[*Φ*(*s,t*)]d*s*. In cases where *R*(*s,t*) = *R*(*s*), i.e. the reward kernel can be appropriately assumed, fixed over the duration of study, *Φ*(*s,t*) takes a simpler form of *tR*(*s*). In the following analysis, we focus on this special case in which, after the shift, the same reward kernel is assumed valid over the ensuing time period/scale under consideration.

**Figure 1.**
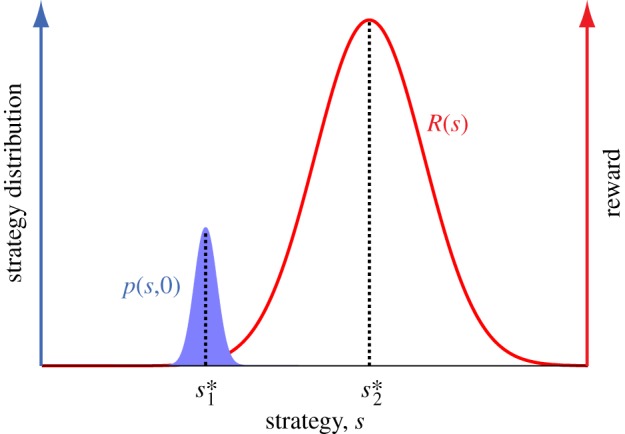
Schematic of the shift-and-response scenario: the red curve represents the reward kernel under the new regime; s*_1_ and s*_2_ are the best strategies, i.e. those with maximum reward, under the old and new regimes, respectively; the blue curve represents the strategy distribution at the time that the shift occurs. The focus of this analysis is on how the strategy distribution responds under the new regime.

Figure 1 illustrates the system we study schematically. We assume that the initial distribution of strategies within the population *p*_0_(*s*) centres about the best strategy under the previous long-standing regime, s*_1_. Then, at some instant *t* = 0, the regime, characterized by reward kernel *R*(*s*), shifts such that the best strategy under the new regime becomes s*_2_ (red curve in figure 1). The population then adapts to this new regime and, ultimately, the strategy distribution *p*(*s,t*) moves towards s*_2_.

**Figure 2.**
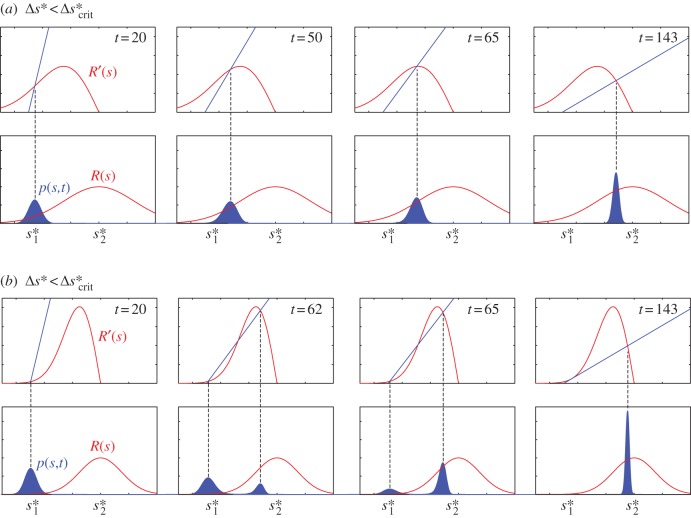
Illustration of the dynamics of the strategy distribution *p*(*s,t*) for a Gaussian-type *R*(*s*): (*a*) travelling peak transition, in which the population move together as a cohesive unit; and (*b*) population-dividing transition, in which *p*(*s,t*) exhibits two peaks during the transition. The initial strategy distribution *p*_0_(*s*)(=*p*(*s*,0)) is the same in both cases. Note that in this particular case, the condition for population dividing is 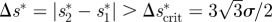 (see text). Therefore, the division can be induced by a small *σ* (as done here) or equivalently a large Δs*. As discussed in the text, *D*^2^ sets the pace of the dynamics: larger *D*^2^ means that the slope of the blue straight line flattens more rapidly.

However, the manner in which the population moves towards s*_2_ matters, and there are two possibilities. First, the population may change as a cohesive unit towards s*_2_; in that case the strategy distribution *p*(*s,t*) would exhibit a ‘travelling peak’-type behaviour (figure 2*a*). Alternatively, the population may divide itself into two groups—one tending to hold on to the old best strategy s*_1_ and the new emerging one tending to adopt the new best strategy s*_2_—and the latter group eventually dominates owing to their greater reward (figure 2*b*). (Multiple peaks are possible, depending on the reward kernel shape, but the same analysis framework still applies.) This difference may have significant implications for a wide range of systems. For example, such division may lead to serious tension in particular social contexts (e.g. polarization and increased inequality that accompanied the transition from centrally planned to market-based economies [16–18]); or correspond to extinction or replacement of a group of species by another in ecological contexts. We show that the conditions of shifts that induce these two different types of responses can be derived based on the observation that the first type corresponds to strategy distribution *p*(*s,t*) that is always unimodal (i.e. having only one peak at all times) and the second type corresponds to *p*(*s,t*) that is temporarily bimodal (i.e. having two peaks).

## 3. Model analysis

Let *s*_*m,t*_ denote all strategies that satisfy 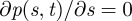 (i.e. 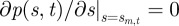); that is, *s*_*m,t*_ locates either a local maximum or a local minimum of the strategy distribution at time *t*. Applying this to the solution given by equation (2.2) yields the following identity for *s*_*m,t*_:3.1
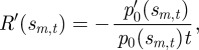
where 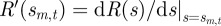 and 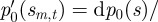 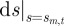. Making a reasonable assumption that the initial strategy distribution *p*_0_(*s*) is well approximated by a Gaussian distribution with mean s*_1_, the formerly best strategy, and with arbitrary variance *D*^2^, presumably maintained by some fluctuation of the reward kernel under the old regime, we obtain a much more useful identity for *s*_*m,t*_:3.2
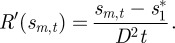


Here, before proceeding to derive further results, some elaboration on the conditions employed in our model development is in order. In particular, we consider the following two conditions: (i) the reward kernel *R*(*s*) is considered fixed over the time scale of the analysis; and (ii) the strategy distribution at the time of shift *p*_0_(*s*) is characterized by variance *D*^2^ presumably maintained by some fluctuation of the reward kernel under the old regime. The central issue here is time scale. Note that if a reward kernel is fixed over a very long time, the model predicts that the strategy distribution would become highly concentrated at its best strategy s*; mathematically, this corresponds to 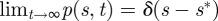. This would imply that *D*^2^ should be very small, on the order of 1/*T*, with *T* being the duration of the old regime. However, over a long time scale, the reward kernel itself would probably exhibit some degree of fluctuation, thereby preventing such concentration of strategies in the population and thus maintaining some diversity of strategies—this diversity is what *D*^2^ represents. Now, in our analysis, the reward kernel *R*(*s,t*) = *R*(*s*) is assumed to be fixed. This is valid only over a relatively short period of time; in fact, the extent to which the reward kernel can be assumed fixed defines the validity of our analytical framework. Note that such validity over a relatively short time scale is consistent with our focus on short-term transient behaviour. Nonetheless, this issue of time scale is important and must be taken into account in future model development and in implementing this model in conjunction with other models.

Equation (3.2) succinctly highlights the importance of strategy diversity at the time of shift: *D*^2^ sets the pace of the population response—larger *D*^2^, faster response—regardless of the division. This is in agreement with existing work in ecology, which emphasizes the importance of biodiversity in coping with environmental changes [19], and with models in mathematical finance in which herding behaviour (reduction of diversity) makes financial markets more fragile [20–22].

Importantly, equation (3.2) allows for a simple, yet powerful and versatile, graphical method that can be used to study the population division and is applicable for reward kernels of arbitrary shape: *s*_*m,t*_ is located where function *R*′(*s*)–(*s*−s*_1_)/*D*^2^*t* changes sign (for a continuous *R*′(*s*), this is simply the intersection between the straight line (*s*−s*_1_)/*D*^2^*t* and *R*′(*s*)). As discussed later, population division corresponds to the distribution of strategies adopted by the population, *p*(*s,t*), having more than one peak. This occurs when there are at least three values for *s*_*m,t*_. It then follows that a necessary condition for the population division is non-concavity of *R*′(*s*), as there are at most two intersections between a straight line and a concave function. It is worth pointing out that while non-concavity has been shown to be responsible for multiple *equilibria* in many ecological [3,4,19,23] and economic [24–27] models, the present analysis addresses something different, namely its effects on *transient* behaviour.

The identity in equation (3.2) is the key in arriving at one of our central findings: the reward kernel-dependent threshold of the shift magnitude that separates cohesion and division of population response. Using equation (3.2) and some geometric arguments (see appendix A and figure 4 therein), it can be shown that the population will respond to the shift by dividing into groups, if3.3
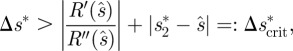
where Δs* = |s*_2_–s*_1_| is the shift magnitude. 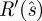 and 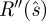 are the first and second derivatives of the reward kernel *R*(*s*), respectively, evaluated at 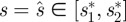. Here 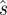 is the closest point to s*_2_ that satisfies 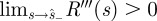 and 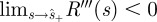 (i.e. 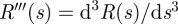 changes sign at 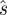), assuming here s*_2_ > s*_1_. (Note that for s*_2_ < s*_1_, these conditions become 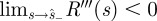 and 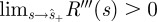 for the same geometrical reason.) For a continuous *R*′(*s*), 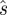 is simply the inflection point of *R*′(*s*). We call the critical value Δs*_crit_ in the above inequality the ‘population-dividing threshold’. Furthermore, equation (3.2) can also be used to calculate the times at which the new peak starts to form and when the old peak completely disintegrates (see appendix B). In addition, it is important to note that while equation (3.3) can be applied to a wide range of families of reward kernels (see table 1 for examples), for very irregular reward kernels (e.g. those involving multiple local maxima, discontinuities, and thus undefined higher-order derivatives of *R*(*s*)), one must resort to equation (3.2) to determine Δs*_crit_.

**Table 1.** Population-dividing threshold Δs*_crit_ for selected reward kernel *R*(*s*). Δs*_crit_, when it exists, is simply proportional to a measure of how wide *R*(*s*) is; this holds even for a heavy-tailed *R*(*s*), such as the Cauchy-type reward kernel, whose variance does not exist. *A,B* and *C* are constants (*B,C* > 0). n.a. indicates that the reward kernel intrinsically does not induce population-dividing transition.

*R*(*s*)		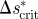
*C* exp{– (*s*–s*_2_)^2^/2*σ*^2^}	(Gaussian)	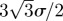
*C* exp{–| *s*–s*_2_|/*σ*}	(exponential, two-sided)	*σ*
*C* {(*s*–s*_2_)^2^ + *a*^2^)}^−1^	(Cauchy, two-sided)	2*a*
*A*–*B*(*s*–s*_2_)^2^	(inverted parabola)	n.a.
*A*–*B*|*s*–s*_2_|	(linear)	n.a.

## 4. Discussion

A few illustrative examples are given in order to demonstrate the significance and applicability of these results. Let us consider two reward kernels with very different shapes, corresponding to different social/ecological regimes. First, we consider *R*(*s*) = *C* exp[–(*s*–s*_2_)^2^/2*σ*^2^], where *C* > 0 is a constant (but not 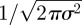 as *R*(*s*) is *not* necessarily a pdf) and *σ* is a parameter representing the width of the kernel. We refer to this as the Gaussian-type (or bell-shaped) reward kernel. For this particular reward kernel, 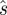 is simply the inflection point of *R*′(*s*), i.e. 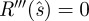, and the population-dividing threshold 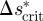 is simply 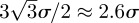 (see appendix A for full derivation and electronic supplementary material, movies S1 and S2). Our analysis suggests that Δs*_crit_, if it exists, is simply proportional to a measure of how wide the reward kernel is; this measure is typically its standard deviation. This statement holds even for those reward kernels whose variance (and standard deviation) does not exist (e.g. the heavy-tailed Cauchy-shape kernel; see table 1).

What real-world situation may be described by a Gaussian-type reward kernel? An important characteristic of the Gaussian-type reward kernel is that even for strategies far away from the best strategy, the marginal change in the reward approaches zero, i.e. the reward kernel is bounded from below. In social contexts, this may correspond to the situations in which there is limited liability or some social safety net that protects against catastrophic losses. Limited liability changes the curvature of the reward function (generally assumed to be concave in economics and finance) because rewards can only fall minimally (or stay constant) below the level at which limited liability binds [28–30].

We contrast this with an alternative situation in which the reward continues to decline significantly for strategies increasingly far away from the best strategy, and agents can experience enormous losses; an example includes financial markets with complex instruments. To capture this reward structure, we consider an inverted parabola reward kernel: *R*(*s*) = *A*–*B*(*s*–s*_2_)^2^. In this case, it can be shown that the strategy distribution *p*(*s,t*) maintains its initial Gaussian shape throughout the transition with time-dependent mean (s*_1_ + 2*BtD*^2^s*_2_)/(1 + 2*BtD*^2^) and variance *D*^2^/(1 + 2*BtD*^2^) (see appendix C and electronic supplementary material, movie S3); that is, the population never splits into two under an inverted parabola *R*(*s*). Thus, there exist reward kernels, such as strictly concave kernels, that intrinsically do not induce population-dividing transient responses (table 1).

**Figure 3.**
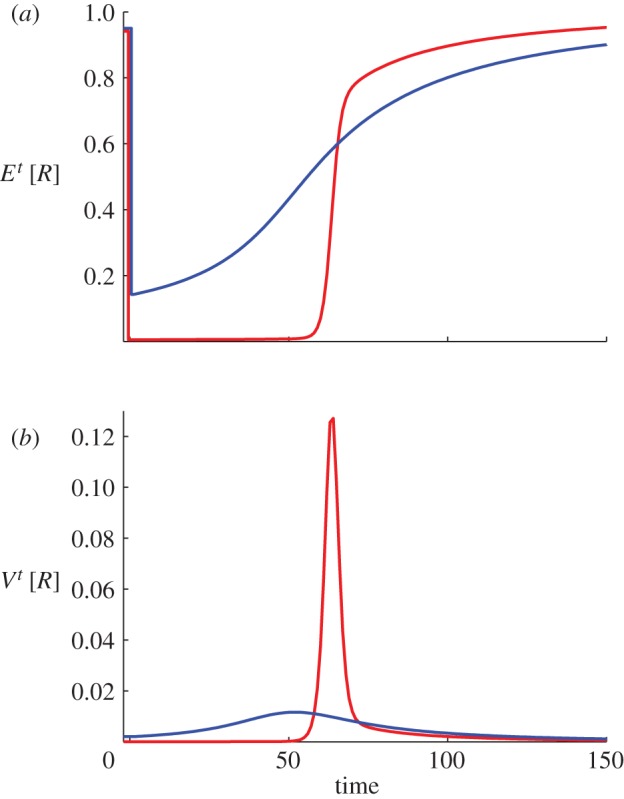
The transient dynamics of the population-averaged reward *E*^*t*^ [*R*] and the variance of reward earned by the population *V*^*t*^[*R*]. Blue lines correspond to the cohesive, travelling-peak, transition (figure 2*a*) and red lines the population-dividing one (figure 2*b*).

**Figure 4.**
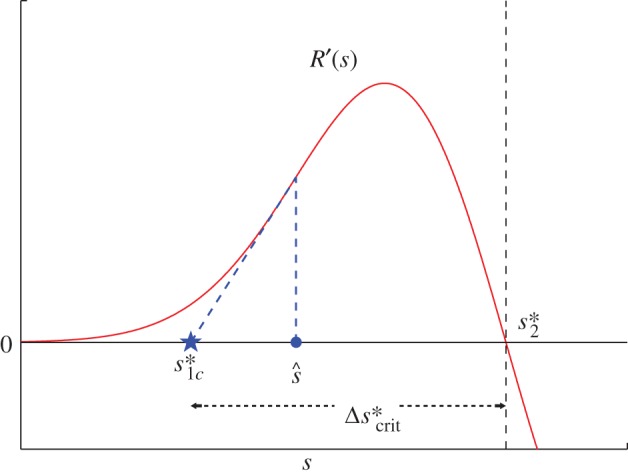
Illustration of the population-dividing threshold for a Gaussian reward kernel. The figure illustrates the graphical arguments used to calculate the population-dividing threshold Δs*_crit_ for the Gaussian reward kernel. If Δs* = |s*_2_–s*_1_| < Δs*_crit_, the pdf of strategies, *p*(*s,t*), will be unimodal at all times, and it will move as a travelling peak in response to the shift in the reward kernel. If Δs* > Δs*_crit_, *p*(*s,t*) will be bimodal for a period of time, corresponding to the population dividing scenario. See figure 2 and the electronic supplementary material, movies S1 and S2. (Online version in colour.)

The difference between the cohesive and population-dividing transitions can also be seen in the dynamics of the population-averaged reward *E*^*t*^[*R*] and the variance of reward earned by the population *V*^*t*^[*R*] (figure 3). Here, we consider again the two shifts with Gaussian-type reward kernels shown in figure 2. In the travelling peak transition, *E*^*t*^[*R*] shows significant improvement immediately after the shift (figures 2*a* and 3*a*). In contrast, in the population-dividing case, *E*^*t*^[*R*] remains low for an extended period of time—as if the population is still in shock due to the shift—and shows a dramatic increase only after the new peak near s*_2_ starts to form (figures 2*b* and 3*a*). This rapid improvement in *E*^*t*^[*R*], however, is accompanied by a large spike of reward inequality, captured by *V*^*t*^[*R*] (figure 3*b*). Note that, in both cases, there is a temporary elevated level of reward inequality; this result suggests that avoiding an increase in inequality is more difficult than avoiding the division of population. Interestingly, similar variance dynamics has also been observed in some studies of long-term response of traits to shifts in selection pressure in genetics literature (see [31] and the references therein). Furthermore, figure 3*b* also suggests that maximum level of inequality would be considerably less and arrive sooner in the cohesive transition than in the population-dividing one.

Finally, we consider some evidence of these patterns in real-world cases. While the empirical data may not be readily available to examine these transient dynamics quantitatively, some historical examples exist that are indicative of the patterns discussed earlier. A major example is the transition of centrally planned economies to market-based economies. Two widely debated aspects related to the design of reforms to bring about this transition are particularly relevant here. The first related to whether the reform process should be carried out quickly in one big stroke (often referred to as ‘shock therapy’ or ‘big-bang’ approach) or in a gradual manner. Proponents of the shock therapy [16,32] pointed to the complementarity of reform measures and thus the need for carrying out the reforms in one decisive stroke. Proponents of the gradualist approach [17,33,34], on the other hand, emphasized the importance of proper sequencing of reform measures. The second major design aspect related to whether safety nets should be introduced given that the reform process was expected to be a risky and highly painful process.

As discussed earlier, the introduction of safety nets makes the reward kernel non-concave and more akin to the Gaussian type. Our results show that while such a reward kernel protects against catastrophic losses, it also opens up the possibility of a division in population. Accordingly, our model predicts that such possibility would be higher under the big-bang approach (owing to its larger magnitude of shift) than the gradualist approach. This resonates with emerging research on the reform processes in several countries. Although the big-bang approach was advocated, in part, on grounds of political expediency, almost the exact opposite happened: political support for the transition was found to be seriously deficient as the reforms progressed. It was pointed out in the study of Dewatripont & Roland [17, p. 1208] that ‘all of the big-bang programs in Eastern Europe have undergone substantial modifications, rejections, or delays’ because of divisions within the population. They cite, in particular, the case of Slovakia, which broke away from Czechoslovakia, and that of Russia, where there was a popular backlash against the reforms. Lithuania and Poland saw the return of former communists to power who seemed to accept the move towards capitalism but at a more gradual pace. At the other end of the spectrum, Hungary and China are often cited as examples of a more gradual transition. In both countries, population movement has been more cohesive, economic performance has been higher and there has been a slower rise in inequality than in the big-bang countries that witnessed more divisive population movements [33]. These features related to the different paths towards reform are generally in agreement with those related to the population-dividing versus cohesive transitions predicted by the model (figure 3).

It is interesting to note that proponents of the big- bang approach drew their inspiration from the experience of West Germany, which had succeeded in rebuilding and reforming its economy following the big-bang approach. However, West Germany reformed under very different conditions following the Second World War. At that time, the idea of safety nets had not become institutionalized and it is probable that the reward kernel for West Germany more closely resembled the inverted parabolic form, for which, according to our model, population movement would be like a cohesive travelling peak.

These examples reinforce the importance of studying transient dynamics. Population-dividing transition can lead to an increase in inequality and violence, which can threaten the viability of reforms or lead to a policy reversal, as in the case of some of the big-bang East European countries. Needless to say, these simple examples do not capture the complexity of the reform process nor the outcomes that followed from it. Our objective in presenting these is to shed light on some commonly observed patterns, and, in the process, raise new questions and directions for future research.

## 5. Conclusion

In sum, we have shown that for some types of exogenous shifts, the population responds together as a cohesive unit, while for others the population responds by dividing into distinct groups. Our analysis suggests that (i) the shape of the reward kernel exerts strong control on the transition dynamics; (ii) the population-dividing threshold Δs*_crit_, when it exists, is simply proportional to a measure of how wide the reward kernel is; and (iii) larger strategy diversity at the time of shift leads to faster response. These results could contribute to a better understanding of the transient dynamics under a wide range of regime shifts observed in human and natural systems, offering guidelines for anticipating population responses to changes (e.g. ecosystem responses to global climate change or social responses to rapid political or economic change) and designing policy to help manage such transitions in order to avoid undesirable outcomes. For example, our analysis shows that limited liability designed to protect against catastrophic losses may induce population division. In several real-world cases, such population divisions have led to conflicts (as in the transition from centrally planned to market economies), which, in turn, have made the transition very costly to navigate and jeopardized the very chances of actually reaching the new equilibrium. We hope that this work will encourage future empirical studies to explore these aspects in greater detail.

## Figures and Tables

**Figure 5.**
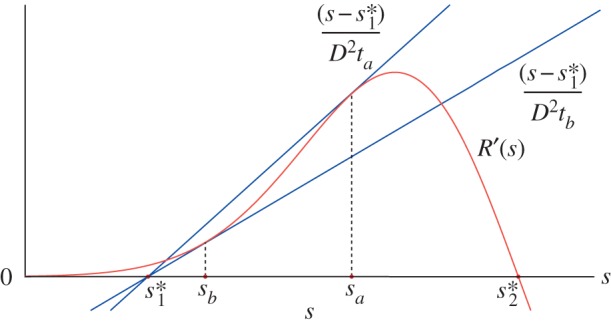
Illustration of the time of formation of the new peak (*t*_*a*_) and the time of disintegration of the old one (*t*_*b*_) for a Gaussian reward kernel. The plot shows the first derivative of the Gaussian reward kernel, *R*′(*s*), and the two straight lines that intersect the curve at one point and are tangent to it at another point. At *t* = *t*_*a*_, a new peak of the strategy distribution *p*(*s,t*), corresponding to the emergence of subpopulation using a new strategy, starts to form, but coexists with the old peak which starts to shrink and disappears completely at *t* = *t*_*b*_. This scenario is when the difference between the two strategies is larger than the population-dividing threshold Δs* > Δs*_crit_. (Online version in colour.)

**Figure 6.**
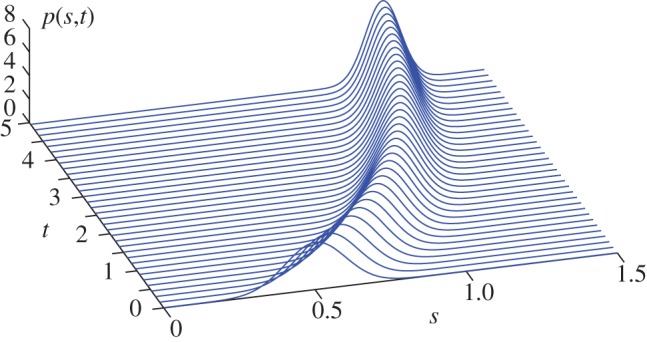
The temporal evolution of strategy pdf, *p*(*s,t*), with an inverted parabola reward kernel. The pdf of strategies *s* at time *t*, i.e. *p*(*s,t*), in the case of an inverted parabola reward kernel with *B* = 20 and s*_1_ = 0.5, and *p*_0_(*s*) being a Gaussian distribution with standard deviation *D* = 0.1 and mean *μ* = 0.5. As 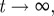 the mean approaches s*_2_ = 1.5 and the variance approaches zero, i.e. 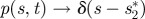. (Online version in colour.)

## Appendix A. Calculation of the ‘population-dividing threshold’ Δ*s**_crit_ for a Gaussian-type reward kernel

In this appendix, we demonstrate how equations (3.2) and (3.3) are applied to a particular type of reward kernel, namely the Gaussian type. Consider a Gaussian-type reward kernel *R*(*s*) = *C* exp[–(*s*–*s**_2_)^2^/2*σ*^2^], where *C*>0 probable is a constant, and assume that *s**_1_ < *s**_2_. Assuming a Gaussian initial pdf of strategies yields the following identity of *s*_*m,t*_ (equation (3.2)):

A1

which is a transcendental equation that can be solved either numerically or, in this case, graphically. Figure 4 shows the plot of *R*′(*s*) and the straight line that intersects and is tangent to *R*′(*s*) at , the inflection point of *R*′(*s*). At , the condition that  and  is satisfied. This straight line passes through the abscissa at *s**_1_ = *s**_1*c*_. It turns out that, if *s**_1_ > *s**_1*c*_, corresponding to a relatively small shift, the straight line will cross the curve of *R*′(*s*) at only one point for all *t* > 0. In contrast, if *s**_1_ < *s**_1*c*_, corresponding to a relatively large shift, the straight line may intersect with the curve of *R*′(*s*) at one or three points depending on the value of time *t* > 0. The number of intersections gives an indication of the dynamics of the pdf of strategies at time *t*, *p*(*s,t*).

To calculate Δ*s**_crit_—the critical shift magnitude that separates between cohesive and divisive transitions of the population—we first determine , the inflection point of *R*′(*s*). This implies that , where  is the third derivative of *R*(*s*) (or the second derivative of *R*′(*s*)). This results in

A2

which has three solutions:  and . As suggested by figure 4,  is the required solution because it lies between *s**_1*c*_ and *s**_2_. Therefore, we obtain

A3

as shown in equation (3.3). Substituting the values of  and   in equation (A3), we obtain

A4

the population-dividing threshold for a Gaussian-type reward kernel.

## Appendix B. Calculation of the time of formation of the new peak and the time of disintegration of the old one for a Gaussian reward kernel

Consider the population-dividing scenario where the difference between the old (*s**_1_) and the new (*s**_2_) best strategies is larger than the population-dividing threshold, i.e. Δ*s** = |*s**_2_–*s**_1_| > Δ*s**_crit_. As illustrated in figure 5, there are two instants of time, *t*_*a*_ and *t*_*b*_, where the straight line intersects *R*′(*s*) at one point and is tangent to it at another. For any time *t* < *t*_*a*_, there is only one intersection, corresponding to *p*(*s,t*) having one peak centred around a strategy close to *s**_1_. On the other hand, for any time *t* > *t*_*b*_ there is also only one intersection, corresponding to *p*(*s,t*) having one peak centred around a strategy close to *s**_2_. For *t*_*a*_ < *t* < *t*_*b*_, the straight line intersects *R*′(*s*) at three different points. During this time, *p*(*s,t*) is bimodal, having one peak close to *s**_1_ and the other close to *s**_2_. In sum, at *t* = *t*_*a*_, the new peak starts to form, and at *t* = *t*_*b*_, the old one disappears.

To calculate the values of *t*_*a*_ and *t*_*b*_, we assume two corresponding points *s*_*a*_ and *s*_*b*_, where the slope of the tangent to *R*′(*s*) at these two points (i.e. *R*^′′^(*s*_*a,b*_)) is equal to that of the straight line (i.e. 1/*D*^2^*t*_*a,b*_),

B1

Substituting for the Gaussian reward kernel ), we obtain

B2

To calculate the values of *s*_*a*_ and *s*_*b*_, we notice from figure 5 that

B3

with  (i.e. *s*_*a,b*_ are not the inflection point of *R*′(*s*)). Substituting for *R*′(*s*_*a,b*_) and  in equation (B 3), we obtain

B4

Let , Δ*s** = *s**_2_–*s**_1_, and assuming that *s**_2_ > *s**_1_ (as shown in figure 5), equation (B 4) can then be written as follows:

B5

Equation (B 5) has two real solutions of *s*_*a*_ and *s*_*b*_ that correspond to *t*_*a*_, *t*_*b*_ > 0, respectively, and one (undesired) imaginary solution that corresponds to some *t* < 0. Note that  and . (Note also that it is possible to derive closed-form expression of *s*_*a*_, *s*_*b*_, *t*_*a*_ and *t*_*b*_, but they are too lengthy and cumbersome to offer any useful insights, and thus not shown here.)

## Appendix C: Exact formula of the probability density function of strategy, *p*(*s*,*t*), for an inverted parabola reward kernel

Consider the inverted parabola reward function *R*(*s*) = *A*–*B*(*s*–*s**_2_)^2^, where  and *B* > 0. Applying the *s*_*m,t*_ identity in the main text, i.e.  , we obtain

C1

which can be solved for *s*_*m,t*_ to get

C2

Equation (C 2) indicates that the probability distribution function of strategy *s* at time *t*, *p*(*s,t*), has only one maximum (i.e. unimodal) at all times, and hence the travelling peak is the only scenario for an inverted parabola reward kernel. Notice that at *t* = 0, *s*_*m,t*_ = *s**_1_ and as , , as expected. This result can also be deduced graphically considering that the left-hand side of equation (C 1) represents a straight line of a negative slope while the right-hand side represents a straight line of a positive slope. The two lines will intersect only at one point.

To write the corresponding *p*(*s,t*), we first calculate *Φ*(*s,t*), and *Z*(*t*) (as defined in the main text)

C3

and

C4

Expanding the brackets and rearranging the terms, equation (C 4) becomes

C5

With some changes of variables, equation (C 5) becomes

C6

where  and .

Completing the square of the exponential term in the integral, equation (C 6) becomes

C7

where we used the fact that  . Substituting the obtained values of *Φ*(*s,t*) and *Z*(*t*) in the solution of the replicator equation, i.e. , we obtain

C8

Expanding the brackets and rearranging the terms, we obtain

C9

Therefore, we have shown that for an inverted parabola reward kernel, *p*(*s,t*) is simply a Gaussian distribution with time-dependent mean () and variance (). Figure 6 illustrates the temporal evolution of *p*(*s,t*) for an inverted parabola reward kernel.
